# TNF-α-induced protein 8-like 2 negatively regulates the immune function of dendritic cells by suppressing autophagy via the TAK1/JNK pathway in septic mice

**DOI:** 10.1038/s41419-021-04327-x

**Published:** 2021-10-30

**Authors:** Shuang-Qing Liu, Chao Ren, Ren-Qi Yao, Yao Wu, Ying-Yi Luan, Ning Dong, Yong-Ming Yao

**Affiliations:** 1 Department of Emergency, the Fourth Medical Center of the Chinese PLA General Hospital, 100048 Beijing, People’s Republic of China; 2 Translational Medicine Research Center, Medical Innovation Research Division and Fourth Medical Center of the Chinese PLA General Hospital, 100048 Beijing, People’s Republic of China; 3 Department of Burn Surgery, the First Affiliated Hospital of Naval Medical University, 200433 Shanghai, People’s Republic of China

**Keywords:** Cell death and immune response, Inflammatory diseases

## Abstract

Tumor necrosis factor (TNF)-α-induced protein 8-like 2 (TIPE2) is a newly discovered negative immunoregulatory protein that is involved in various cellular immune responses to infections. However, the underlying mechanism by which TIPE2 affects the immune function of dendritic cells (DCs) is not yet understood. This study aimed to determine the correlations among DCs TIPE2 expression, autophagic activity and immune function in the context of sepsis. In addition, the signaling pathway by which TIPE2 regulates autophagy in DCs was investigated. We reported for the first time that TIPE2 overexpression (knock-in, KI) exerted an inhibitory effect on autophagy in DCs and markedly suppressed the immune function of DCs upon septic challenge both in vitro and in vivo. In addition, TIPE2 knockout (KO) in DCs significantly enhanced autophagy and improved the immune response of DCs in sepsis. Of note, we found that the transforming growth factor-β (TGF-β)-activated kinase-1 (TAK1)/c-Jun N-terminal kinase (JNK) pathway was inhibited by TIPE2 in DCs, resulting in downregulated autophagic activity. Collectively, these results suggest that TIPE2 can suppress the autophagic activity of DCs by inhibiting the TAK1/JNK signaling pathway and further negatively regulate the immune function of DCs in the development of septic complications.

## Introduction

Sepsis is still a nagging medical syndrome that occurs when a serious infection exceeds the host’s scavenging ability and leads to systemic inflammatory response syndrome. Sepsis is a common complication derived from complicated infection, trauma, shock, and major surgery [1–3]. As a heavy burden on national health and the economy, sepsis is a leading cause of hospital death in intensive care units (ICUs) worldwide [4–6]. The interaction between excessive inflammation and immune suppression determines septic outcomes [7, 8]. Substantial progress in the clinical understanding and physiopathological mechanism obviously improves patient outcome, but the mortality due to sepsis is still very high, ranging from 14.7 to 29.9% [5, 9–11].

Dendritic cells (DCs) play pivotal roles in coordinating aberrant immunity as the link between the innate and adaptive immune systems [12, 13]. DCs are representative antigen-presenting cells that initiate the immune response by capturing, processing, and transporting antigens from somatic to secondary lymphoid tissues [14, 15]. Emerging evidence suggests that widespread DC apoptosis [16, 17] and dysfunction [12, 18–20] can lead to immunosuppression associated with sepsis.

Macroautophagy (referred to hereafter as autophagy) is an important process of intracellular material turnover in eukaryotes. The link between the immune function of DCs and autophagy is extensive and includes DC maturation, migration, antigen presentation, cytokine production, and T-cell activation [21]. Therefore, autophagy might be involved in the induction of immunological paralysis by affecting the abnormal immune response of DCs.

Tumor necrosis factor (TNF)-α-induced protein 8-like 2 (TIPE2, or TNFAIP8L2) is a recently discovered negative regulatory protein. Depletion of TIPE2 was reported to be related to fatal inflammatory diseases in TIPE2-deficient mice [22]. Moreover, TIPE2 knockout (KO) mice exhibited resistance to bacterial infection via enhanced innate immunity [23]. Furthermore, our previous study showed that the expression of TIPE2 was negatively correlated with the immune function of DCs in the context of burn injury or sepsis [24].

In this study, we investigated the potential impact of TIPE2 on DC autophagy and further examined the underlying pathway by which TIPE2 regulates autophagy, which will promote a better understanding of the molecular mechanism of DC immune dysfunction.

## Materials and methods

### Animals

Experiments were performed on 6-week-old male C57BL/6J mice (weight range 20–25 g), which were provided by the Institute of Laboratory Animal Science, Peking Union Medical College, Beijing, China. TIPE2 gene knockout (TIPE2^−/−^) and knock-in (KI, TIPE2^+/+^) mice on a C57BL/6J background were constructed by the Nanjing Biomedical Research Institute of Nanjing University, Nanjing, China. Animals were randomly divided into different experimental groups or control groups. All mice were maintained with a 12-hour (h) dark-light cycle at a temperature. The animals were provided with laboratory chow and water ad libitum.

### Generation of TIPE2^−/−^ and TIPE2^+/+^ mice

The TIPE2^−/−^ model was created by CRISPR/Cas9 (D10A)-mediated genome engineering. To establish the TIPE2^+/+^ model, the *H11-CAG-Tnfaip812-P2A-EGFP-polyA* knock-in mouse model was generated via the CRISPR/Cas9 system.

### Reagents

CD4 (L3T4) microbeads, CD11c (N418) microbeads, and phycoerythrin (PE)-Cyanine 5-conjugated anti-mouse major histocompatibility complex (MHC)-II were purchased from eBioscience (San Diego, CA). PE-conjugated anti-mouse CD86, allophycocyanin (APC)-conjugated anti-mouse CD80, and fluorescein isothiocyanate (FITC)-conjugated goat anti-mouse CD11c were purchased from Miltenyi Biotec (GmbH, Bergisch Gladbach, Germany). SQSTM1/p62, Beclin-1, LC3B, c-Jun N-terminal kinase (JNK), phospho-JNK, transforming growth factor-β (TGF-β)-activated kinase-1 (TAK1), and phospho-TAK1 antibodies were purchased from Cell Signaling Technology (Danvers, MA).

### Cecal ligation and puncture (CLP) model

After anesthesia, the mice were fixed and the abdominal area was disinfected. An incision was made along the midline of the abdomen, and then the peritoneum was opened to expose the cecum. The cecum was ligated in the middle and punctured twice. Sham-operated mice underwent the same surgical procedure without the ligation and perforation steps.

### Isolation of splenic DCs and T lymphocytes

Under aseptic conditions, murine spleens were removed from the abdominal cavity. Mononuclear cells were isolated from the spleens, lymphocytes were isolated using a CD4^+^ T-cell isolation kit (Miltenyi Biotec GmbH, Bergisch Gladbach, Germany), and DCs were isolated using a CD11c^+^ dendritic cell isolation kit (Miltenyi Biotec GmbH, Bergisch Gladbach, Germany).

### RNA interference

Small interference RNA to Beclin-1 (siRNA) or overexpression RNA to Beclin-1 (LV RNA) was generated by Genchem Co., Shanghai, China. DCs were introduced with recombinant lentiviruses that carry the Beclin-1-siRNA or Beclin-1-LV RNA. Transfection of recombinant lentiviruses was performed according to the manufacture’s instruction.

### Flow cytometric analysis of costimulatory molecules on DCs

DCs (5 × 10^5^) in 100 μl of PBS supplemented with 5% FCS and 0.1% sodium azide were incubated for 30 min at 4 °C with antibodies of MHC-II, CD80, and CD86. Then, the cells were fixed with 1% paraformaldehyde or analyzed by flow cytometry within 1 h using a FACScan (BD Biosciences, Mountain View, CA).

### Laser-scanning confocal microscopy

Cells (2 × 10^6^) were harvested and washed with PBS. Then, the cells were permeabilized with 0.5% Triton X-100 or digitonin and fixed with 4% formaldehyde. The cells were blocked with 5% bovine serum albumin for 1 h and incubated with primary antibodies overnight and with FITC- and PE-conjugated antibodies for 1 h. Images were acquired under a fluorescence microscope.

### Transmission electron microscopy

DCs were fixed for 1 h at room temperature. The samples were embedded in epoxy resin after being dehydrated through a graded ethanol series. Ultrathin sections were prepared and stained. Autophagosomes were identified by using a Hitachi H-7500 transmission electron microscope (Hitachi, H-7500, Japan).

### Western blotting analysis

Blotting was performed with primary antibodies against TIPE2, LC3-I/II, p62, Beclin-1, TAK1, p-TAK1, JNK, p-JNK, and β-actin overnight, followed by incubation with secondary antibodies conjugated with horseradish peroxidase. Immunoreactivity was visualized by an ECL detection system (Amersham Biosciences, Uppsala, Sweden).

### Enzyme-linked immunosorbent assay

The interleukin (IL)-12, TNF-α, interferon (INF)-γ, and IL-4 levels were measured by enzyme-linked immunosorbent assay (ELISA) using mouse simple step ELISA kits (Excell Inc., Shanghai, China). The plates were read in a microplate reader (Spectra MR, Dynex, Richfield, MN).

### Carboxyl fluorescein succinyl ester (CFSE) staining

CD3 was added to a 96-well plate and incubated for 120 min. The CD4^+^ T cells were stained with CFSE and incubated in the dark for 20 min. PBS was added to terminate the staining. CD4^+^ T cells were resuspended in medium supplemented with CD28 and incubated for 24 h. DCs were cocultured with CD4^+^ T cells for 72 h.

### Data analysis

The data are presented as the means ± standard deviation. Statistical comparisons were performed using one-way analysis of variance (ANOVA) or Student’s *t* test. In all cases, *P* < 0.05 was considered statistically significant.

## Results

### Dynamic alterations in autophagy in DCs treated with lipopolysaccharide (LPS) in vitro

DCs were treated with 1 μg/ml LPS for 0, 3, 6, 12, and 24 h in vitro. The expression of LC3-II/I, p62, and Beclin-1 was used to measure autophagy levels [25] in DCs. As shown in Supplementary Fig. S1, stimulation with LPS for 12 h markedly decreased levels of p62 and significantly increased LC3-II and Beclin-1 levels compared with other time points (*P* < 0.05).

### TIPE2 negatively regulates DC autophagy in vitro

**Fig. 1:**
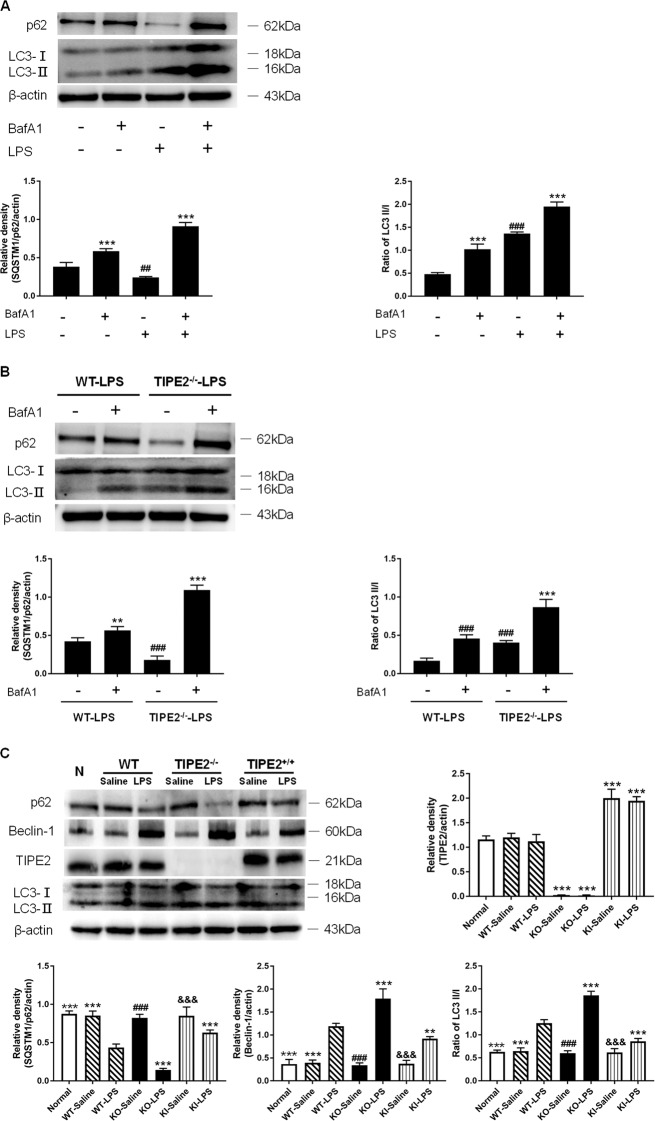
Downregulation of TIPE2 on the autophagic flux of DCs in vitro. **A** Individually or in combination, controls are untreated cells. Mice splenic DCs were treated with LPS (1 μg/ml) for 12 h. Bafilomycin A1 (100 nM) was pretreated 30 min before LPS exposure. Levels of p62 and LC3-II/I in DCs were assessed by western blotting. **B** DCs were isolated from wild-type (WT) and TIPE2^−/−^ (KO) mice spleens with the same dose of LPS (1 μg/ml) for 12 h in the absence or presence of Bafilomycin A1 (100 nM). Levels of p62 and LC3-II/I in DCs were assessed by Western blotting. **C** DCs were isolated from three different TIPE2 genetic designs and treated with the same dose of LPS (1 μg/ml) or saline in vitro. DCs in the normal group were isolated from the murine spleens of WT without any intervention served as the blank control group. Levels of TIPE2, p62, Beclin-1, and LC3-II/I were assessed by Western blotting. β-actin served as the internal standard. Values of three independent experiments are represented as mean ± SD (*n* = 5 in each group). Statistical significances: **A**, **B** compared with other groups, ^**^P < 0.01, ^***^P < 0.001; compared with the untreated group, ^##^*P* < 0.01, ^###^*P* < 0.001. **C** Compared with the WT-LPS group, ^**^P < 0.01, ^***^P < 0.001; compared with the KO-LPS group, ^###^*P* < 0.001; compared with the TIPE2^+/+^ (KI)-LPS group, ^&&&^*P* < 0.001.

Autophagosome accumulation can be monitored by the characteristic conversion from endogenous LC3-I to LC3-II, while the inhibition of fusion between autophagosomes and lysosomes can be reflected by increased levels of p62. Bafilomycin A1 (BafA1) is widely used to reduce autophagosome turnover at a late stage [26]. As shown in Fig. 1A, B, BafA1 treatment markedly increased levels of LC3-II/I and p62 both in wild-type (WT) and TIPE2^−/−^ DCs (*P* < 0.01). It was noted that LPS stimulation (1 μg/ml) markedly elevated levels of LC3-II/I and decreased levels of p62 than untreated DCs (*P* < 0.01), and co-treatment with BafA1 dramatically increased levels of LC3-II/I in DCs than single BafA1 or LPS treatment group (*P* < 0.01). Moreover, LPS treatment significantly upregulated levels of p62 instead of downregulation of p62 expression in the presence of BafA1. Together, these results provided strong evidence that LPS increased autophagic flux in DCs in the absence or presence of TIPE2 in vitro.

To further investigate the correlation of TIPE2 and autophagy in DCs after LPS stimulation in vitro, we isolated DCs from murine splenocytes with different TIPE2 expression (WT, TIPE2^−/−^, and TIPE2^+/+^). As shown in Fig. 1C, LPS significantly augmented autophagic activity in WT DCs, as characterized by increased Beclin-1 levels and LC3-II/I ratios, as well as decreased p62 levels, compared with those in the control group (*P* < 0.05). Moreover, obviously enhanced autophagy levels were observed in TIPE2^−/−^ DCs stimulated with LPS (*P* < 0.05), suggesting that TIPE2 could be involved in the regulation of DC autophagy. We further confirmed the alterations in autophagy levels in TIPE2^+/+^ DCs stimulated with LPS, and the autophagic response was markedly inhibited due to TIPE2 overexpression (*P* < 0.05). In the normal saline group, TIPE2 expression did not affect the autophagy levels (*P* > 0.05).

**Fig. 2:**
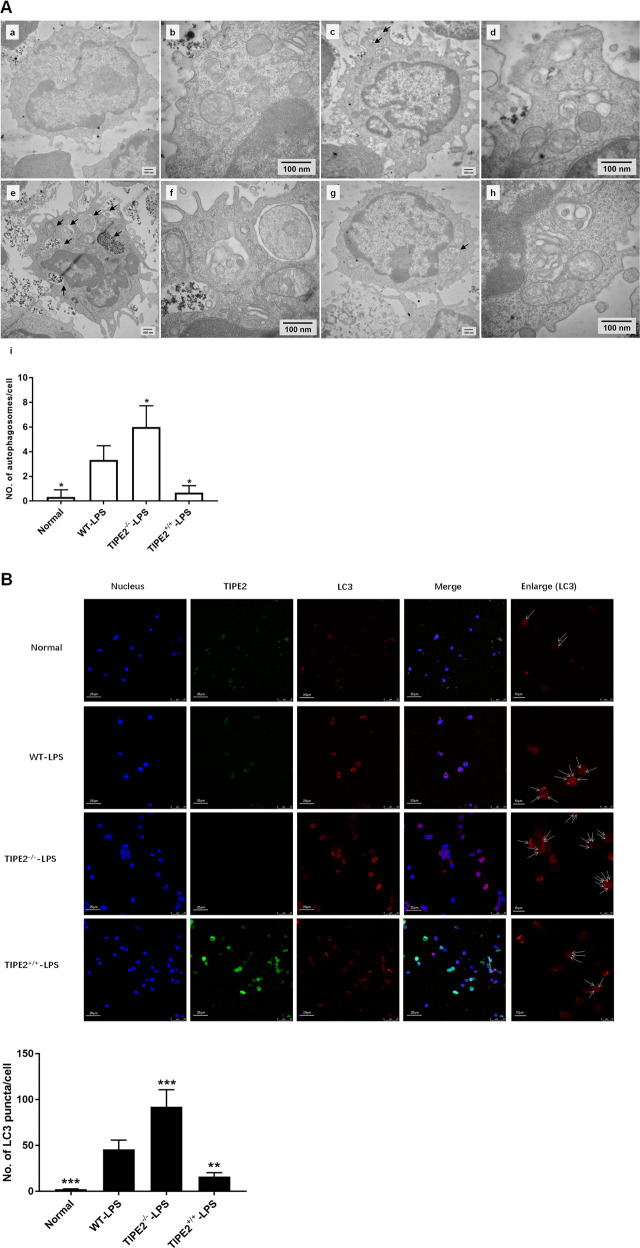
Inhibitory effect of TIPE2 on the formation of autophagosomes in DCs in vitro. **A** Transmission electron microscope was used to assess the autophagosomes in DCs in the four groups (a, b: the normal group; c, d: the WT-LPS group; e, f: the TIPE2^−/−^-LPS group; g, h: the TIPE2^+/+^-LPS group; i: histogram of the number of autophagosomes per cell; a, c, e, g, scale bar=500 nm; b, d, f, h, scale bar = 100 nm), arrows indicated autophagosomes which are defined as a double-membraned structure containing the rem-nants of cellular organelles. **B** Laser-scanning confocal microscopy was used to observe the puncta of LC3. It is shown by anti-TIPE2 antibodies (green) and LC3 (red) using immunofluorescence and confocal microscopy (×600, scale bar = 25 μm; ×1200, scale bar = 10 μm). In addition, the cell nuclei were stained blue by dihydrochloride (DAPI). Data of three independent experiments are represented as mean ± SD (*n* = 6 in each group). Statistical significances: compared with the WT-LPS group, ^*^P < 0.05, ^**^P < 0.01, ^***^P < 0.001.

Transmission electron microscopy is usually used to study autophagy in various types of cells at the ultrastructural level [27]. As shown in Fig. 2A, the number of autophagosomes per cell was significantly increased in the WT-LPS group compared with the normal group in vitro. The density of autophagosomes per cell was further increased in the absence of TIPE2; conversely, the number of autophagosomes in DCs was markedly decreased upon TIPE2 overexpression.

Laser-scanning confocal microscopy was performed to examine the protein expression of TIPE2 (green) and LC3 (red) morphologically in DCs. As shown in Fig. 2B, the number of LC3 puncta in the absence of TIPE2 was obviously increased compared with that of WT DCs following LPS stimulation. Conversely, the number of LC3 puncta per cell was markedly reduced in TIPE2^+/+^ DCs.

### TIPE2 impairs the immune function of DCs by inhibiting autophagy in vitro

**Fig. 3:**
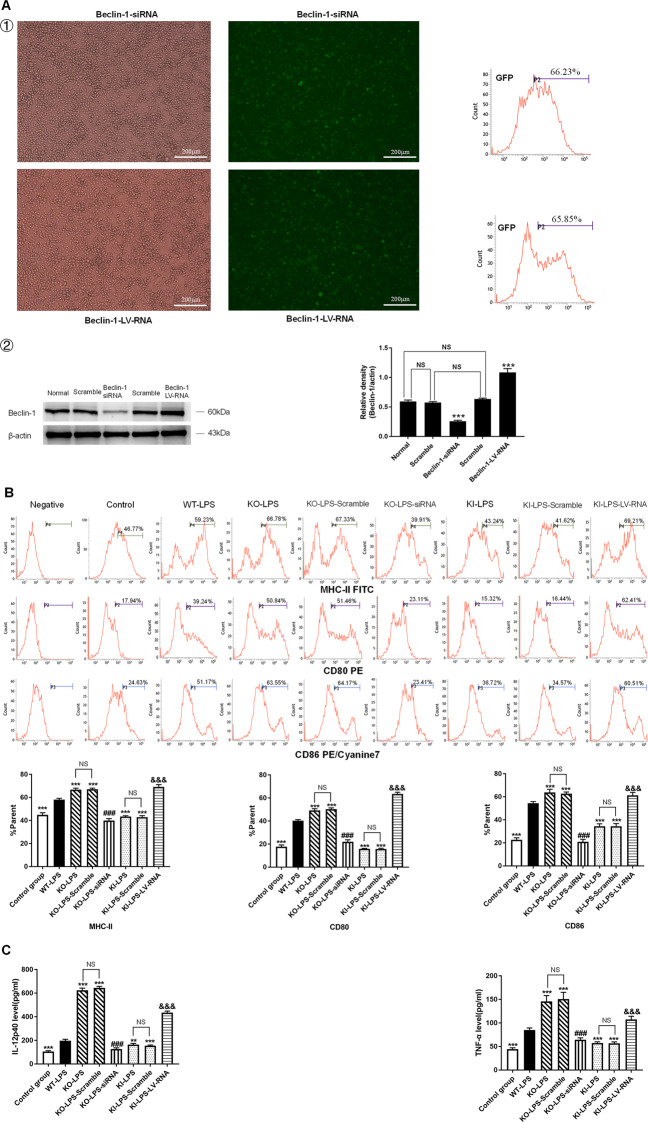

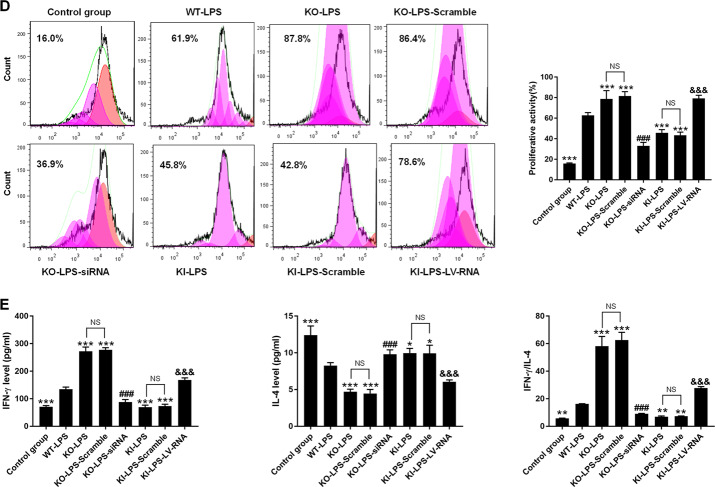
Negative regulation of the immune function of DCs by TIPE2 in vitro. DCs were isolated from murine splenocytes with different TIPE2 genetic designs. DCs were transduced with lentiviral vectors (Beclin-1 knockdown and Beclin-1 overexpression), and scramble cells were transduced with blank vector. **A ①** Transduced cells were analyzed by fluorescence microscopy (×400), scale bar = 200 μm, and then green fluorescence protein (GFP)-positive efficiency was evaluated using flow cytometry. **②** Protein levels of Beclin-1 were determined by western blotting. **B** Representative flow cytometric analysis of CD80, CD86, and MHC-II expressions on DCs in vitro. **C** Expressions of cytokines (IL-12p40 and TNF-α) by DCs were measured by ELISA. **D** Proliferation of CD4^+^ T cells was examined by CFSE staining after incubating with DCs in a 100:1 ratio for 72 h. **E** IL-4 and IFN-γ levels in culture medium were determined by ELISA to evaluate Th1/Th2 polarization. Data obtained from three independent experiments are represented as mean ± SD (*n* = 6 in each group). Statistical significances: **A** compared with the Normal group or the Scramble group, ^***^P < 0.001. **B**–**E** Compared with the WT-LPS group, ^*^P < 0.05, ^**^P < 0.01, ^***^P < 0.001; compared with the KO-LPS group, ^###^*P* < 0.001; compared with the KI-LPS group, ^&&&^*P* < 0.001. NS means no statistical difference between the two groups.

Beclin-1, also called autophagy-related gene (ATG) 6, serves as a pivotal autophagy regulator [28]. Murine splenic DCs were transduced with recombinant lentiviruses that carried Beclin-1 siRNA (knockdown) or Beclin-1-LV-RNA (overexpression) (Fig. 3A ①). It was noted that the expression level of Beclin-1 was downregulated in the Beclin-1 siRNA group and markedly upregulated in the Beclin-1-LV-RNA group, compared with the normal group and scramble group (*P* < 0.001) (Fig. 3A ②).

In this study, we observed that the percentages of DCs expressing the costimulatory molecules CD80, CD86, and MHC-II were significantly downregulated, and DC-induced CD4^+^ T-cell proliferation (Fig. 3B, D) and the production of cytokines, including IL-12p40 and TNF-α (Fig. 3C), were obviously decreased in the KI-LPS group compared with WT-LPS group (*P* < 0.05). An improved DC immune response was noted in the KI-LPS-LV-RNA group compared with the KI-LPS group. Moreover, deletion of the TIPE2 gene strengthened DC function compared with the WT-LPS group and KI-LPS group, and DC function was markedly exacerbated when autophagy was inhibited by Beclin-1 silencing in the TIPE2^−/−^ DCs. Furthermore, coculturing DCs with T cells in the presence of LPS significantly elevated IFN-γ levels and IFN-γ/IL-4 ratios compared with those in the control group, and overexpression of TIPE2 obviously diminished IFN-γ levels and IFN-γ/IL-4 ratios (Fig. 3E), implicating a helper T-cell (Th)2 response to TIPE2 induction. Conversely, deficiency of TIPE2 promoted IFN-γ production in T cells and increased the IFN-γ/IL-4 ratio. IFN-γ levels and IFN-γ/IL-4 ratios in T cells were significantly increased after Beclin-1 overexpression and were downregulated after Beclin-1 knockdown.

### TIPE2 restrains autophagic activity in DCs in vivo

**Fig. 4:**
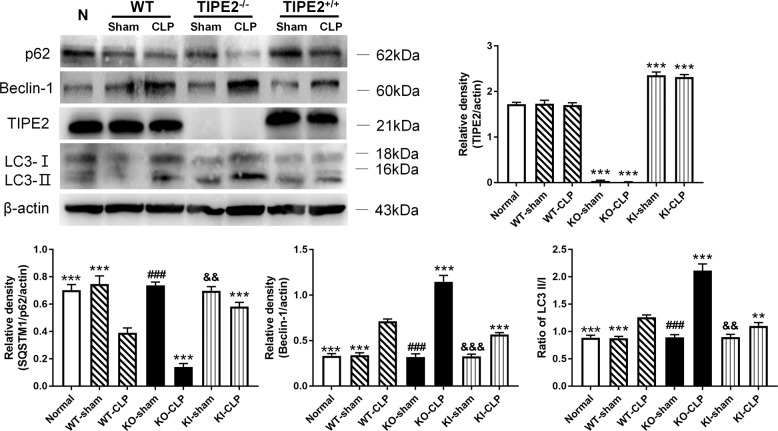
Impact of TIPE2 on autophagy activity of DCs in vivo. DCs were extracted from murine spleens in CLP or sham operation models. DCs from WT murine spleens without any intervention served as the blank control group. Data were obtained by densitometric analysis of western blots of TIPE2, p62, Beclin-1, and LC3-II/I, and β-actin served as the internal standard. Values of three independent experiments were represented as mean ± SD (*n* = 5 in each group). Statistical significances: compared with the WT-CLP group, ^**^P < 0.01, ^***^P < 0.001; compared with the KO-CLP group, ^###^*P* < 0.001; compared with the KI-CLP group, ^&&^*P* < 0.01, ^&&&^*P* < 0.001.

We utilized mice with different TIPE2 genetic backgrounds to establish CLP models. As shown in Fig. 4, the levels of LC3-II/I and Beclin-1 were significantly upregulated, and p62 was downregulated in the TIPE2^−/−^- CLP group compared with the WT-CLP group and the TIPE2^+/+^-CLP group (*P* < 0.05). In contrast, overexpression of TIPE2 obviously dampened autophagy in DCs from the CLP group compared with those from WT-CLP group mice (*P* < 0.05). In the sham operation group, autophagic activity was consistent with that in the normal group.

**Fig. 5:**
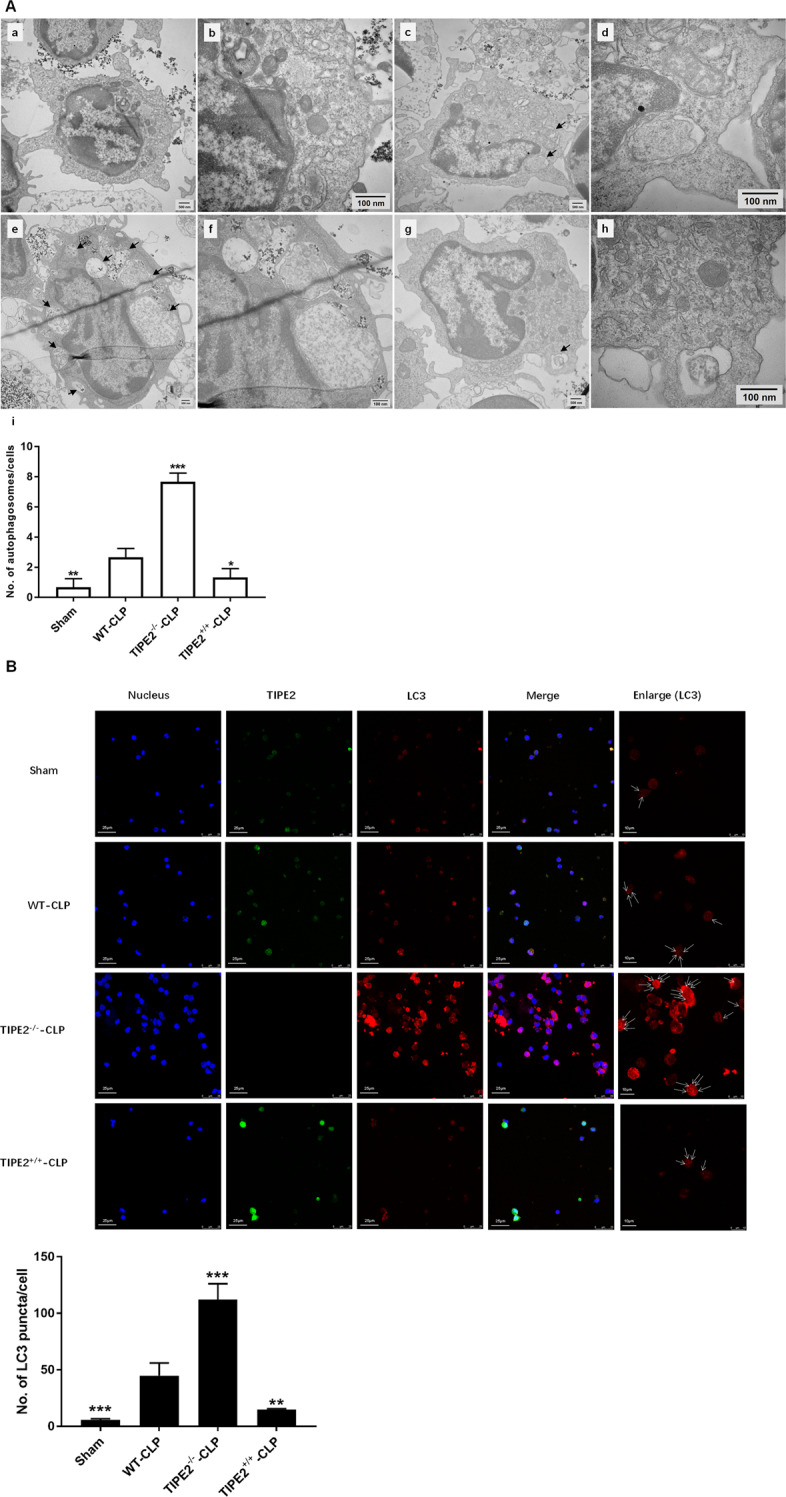
Inhibitory effect of TIPE2 on the formation of autophagosomes in DCs of CLP mice. **A** Transmission electron microscope was used to determine the autophagosomes in DCs in the four groups (a, b: the sham group; c, d: the WT-CLP group; e, f: the TIPE2^−/−^-CLP group; g, h: the TIPE2^+/+^-CLP group; i: histogram of the number of autophagosomes per cell; a, c, e, g, scale bar = 500 nm; b, d, f, h, scale bar = 100 nm), arrows indicated autophagosomes. **B** Laser-scanning confocal microscopy was used to observe the puncta of LC3. It is shown by anti-TIPE2 antibodies (green) and LC3 (red) using immunofluorescence and confocal microscopy (×600, scale bar = 25 μm; ×1200, scale bar = 10 μm). In addition, cell nuclei were stained blue by dihydrochloride (DAPI). Data of three independent experiments are represented as mean ± SD (*n* = 6 in each group). Statistical significances: compared with the WT-CLP group, ^*^P < 0.05, ^**^P < 0.01, ^***^P < 0.001.

The number of autophagosomes in the WT-CLP group was significantly increased compared with that in the sham group (*P* < 0.05, Fig. 5A). The number of autophagosomes was higher in the TIPE2^−/−^-CLP group compared with the WT-CLP group. An obvious decrease in autophagosome formation was observed in the TIPE2^+/+^-CLP group compared with the WT-CLP group (*P* < 0.05). Moreover, LC3 puncta were observed by laser-scanning confocal microscopy to assess autophagic activity. As shown in Fig. 5B, fewer accumulated LC3 fluorescent puncta were detected in the TIPE2^+/+^-CLP group than those in the WT-CLP group (*P* < 0.05). Knockout of TIPE2 caused increased autophagy, as reflected by more LC3 puncta per cell than those in the other two groups (*P* < 0.05).

### Autophagy induction ameliorates TIPE2-mediated immune dysfunction in DCs in vivo

DCs were harvested from spleens 24 h after CLP [29]. We applied the autophagy inhibitor 3-MA and the autophagy inducer rapamycin (Rap) to change autophagy status. In the treatment groups, rapamycin (5 mg/kg) [29, 30] was given by intraperitoneal injection 1 h after CLP operation, and 3-MA (15 mg/kg) [31] was intraperitoneally injected 30 min before CLP operation.

**Fig. 6:**
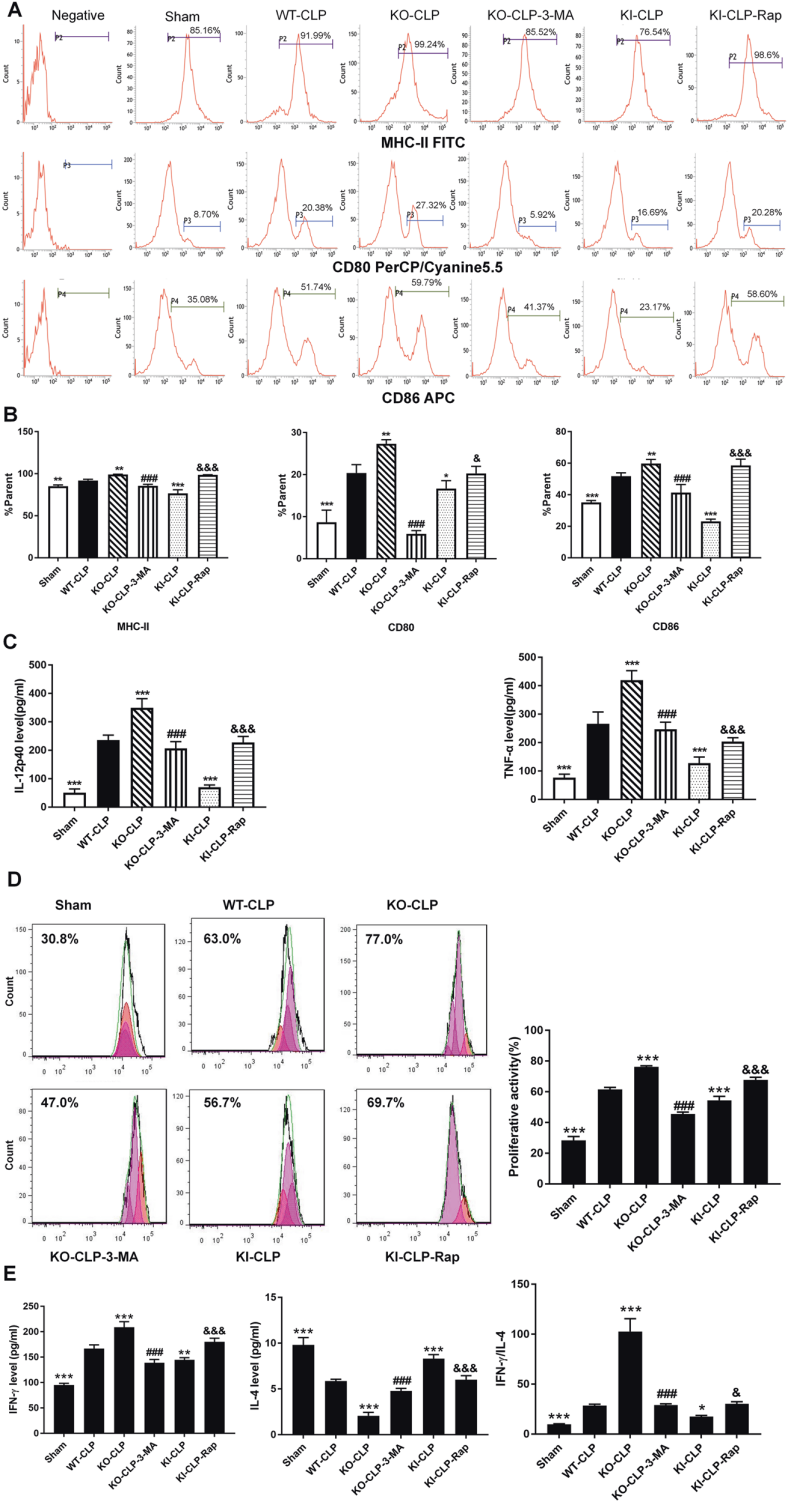
Inhibitory effect of TIPE2 on the immune response of DCs in vivo. DCs were isolated from murine splenocytes in CLP or sham operation models with various TIPE2 genetic designs and divided into six groups. **A**, **B** Representative flow cytometric analysis of CD80, CD86, and MHC-II expressions on DCs in vivo. **C** Release of cytokines including IL-12p40 and TNF-α by DCs was measured by ELISA. **D** Proliferation of CD4^+^ T cells was examined by CFSE staining after incubating with DCs in a 100:1 ratio for 72 h. **E** IL-4 and IFN-γ levels in culture medium were determined by ELISA to evaluate Th1/Th2 polarization. Data of three independent experiments are represented as mean ± SD (*n* = 6 in each group). Statistical significances: compared with the WT-CLP group, ^*^P < 0.05, ^**^P < 0.01, ^***^P < 0.001; compared with the KO-CLP group, ^###^*P* < 0.001; compared with the KI-CL*P* group, ^&^*P* < 0.05, ^&&&^*P* < 0.001.

As shown in Fig. 6A and B, the percentages of DCs expressing costimulatory molecules in CLP group mice were significantly enhanced compared with those in the sham group (*P* < 0.05). The percentage of DCs expressing these molecules was decreased by TIPE2 overexpression (*P* < 0.05). Conversely, the percentages of DCs expressing costimulatory molecules were obviously increased in TIPE2-deficient CLP mice compared with mice in the WT-CLP group (*P* < 0.05). Interestingly, 3-MA treatment reduced the percentage of DCs expressing costimulatory molecules compared with those in the KO-CLP group (*P* < 0.05). Moreover, rapamycin administration increased the expression of costimulatory molecules on DCs compared with those in the KI-CLP group (*P* < 0.05). TIPE2 expression prevented DC-mediated cytokine secretion and CD4^+^ T-cell proliferation (Figs. 6C, D). The abovementioned function could be restored by rapamycin treatment through the promotion of autophagy. Conversely, 3-MA exacerbated damage to DC function and counteracted the effect of TIPE2 knockout. Furthermore, coculturing DCs from WT-CLP mice with CD4^+^ T cells markedly elevated IFN-γ levels and IFN-γ/IL-4 ratios, and these effects were further increased in CD4^+^ T cells in the KO-CLP group (Fig. 6E). In addition, TIPE2 overexpression decreased IFN-γ production in CD4^+^ T cells and downregulated the IFN-γ/IL-4 ratio, indicating a Th2 response. Likewise, IFN-γ levels and IFN-γ/IL-4 ratios in CD4^+^ T cells were significantly increased upon rapamycin exposure and decreased by 3-MA treatment.

### The TAK1/JNK pathway is involved in autophagy induction in DCs

**Fig. 7:**
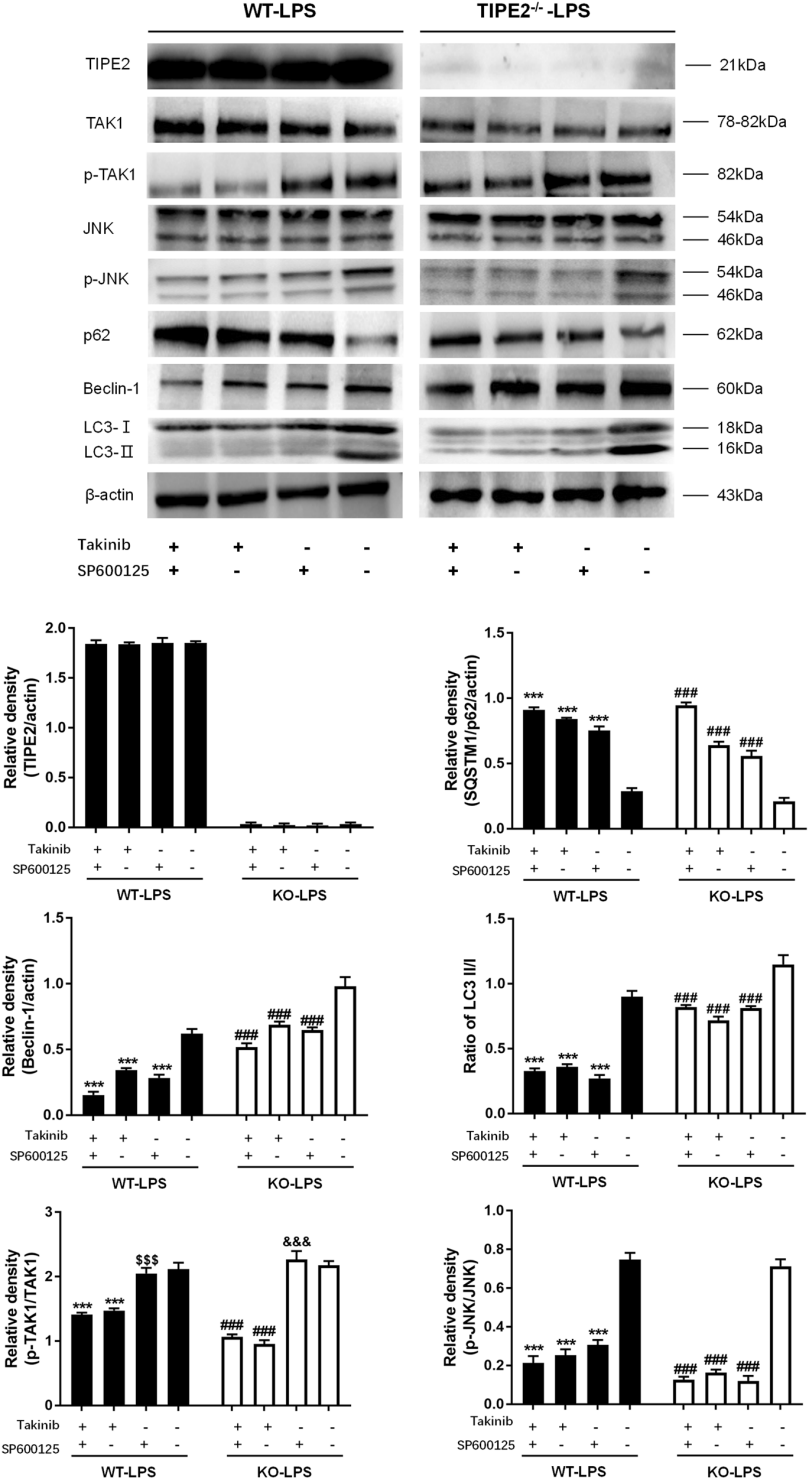
Negative regulation of autophagy by the TAK1/JNK signaling pathway in DCs treated by LPS in vitro. DCs isolated from WT and TIPE2 knockout murine spleens were stimulated by LPS for 12 h. DCs were divided into four groups according to treated inhibitors (Takinib, inhibitor of TAK1; SP600125, inhibitor of JNK). Expressions of TIPE2, p62, Beclin-1, LC3-II/I, TAK1, JNK as well as phosphorylation of both TAK1 and JNK were determined by western blotting, and β-actin served as the internal standard. Values of three independent experiments were represented as mean ± SD (*n* = 5 in each group). Statistical significances: (1) In TIPE2 WT DCs: compared with the group that did not receive any inhibitor, ^***^P < 0.001; compared with that treated with TAK1 inhibitor, ^&&&^*P* < 0.001; (2) In TIPE2 knockout DCs: compared with the group that did not receive any inhibitor, ^###^*P* < 0.001; compared with that treated with TAK1 inhibitor, ^&&&^*P* < 0.001.

DCs were pretreated with Takinib (a potent inhibitor of TAK1 with an IC50 of 9.5 nM) or SP600125 (an inhibitor of JNK1 and JNK2 with an IC50 of 40 nM) and then exposed to 1 μg/ml LPS for 12 h in vitro. As shown in Fig. 7, treatment with Takinib obviously abated the phosphorylation of both TAK1 and JNK, and significantly descending phosphorylation levels of JNK were observed when JNK was inhibited by SP600125, suggesting that TAK1 was an upstream protein of JNK and that TAK1 phosphorylation played a positive regulatory role in the activation of JNK in DCs.

Compared with DCs challenged with LPS, autophagic activity in DCs was obviously diminished when DCs were pretreated with Takinib or SP600125, especially when DCs were pretreated with both inhibitors prior to LPS stimulation, as evidenced by markedly elevated expression of p62 together with reduced expression of LC3-II/I and Beclin-1.

### TIPE2 suppresses DC autophagy by downregulating the TAK1/JNK pathway

**Fig. 8:**
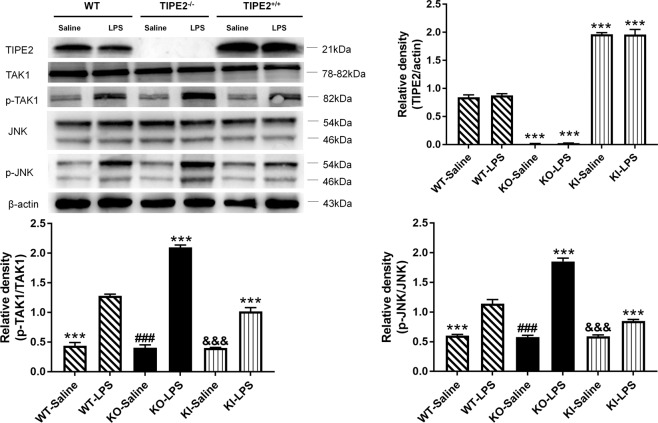
Downregulation of TIPE2 on the TAK1/JNK signaling pathway in DCs in vitro. DCs with different TIPE2 genetic designs were treated by the same dose of LPS or saline in vitro. Expressions of TIPE2, TAK1, JNK as well as phosphorylation of both TAK1 and JNK were determined by western blotting, and β-actin served as the internal standard. Values of three independent experiments are represented as mean ± SD (*n* = 5 in each group). Statistical significances: compared with the WT-LPS group, ^***^P < 0.001; compared with the KO-LPS group, ^###^*P* < 0.001; compared with the KI-LPS group, ^&&&^*P* < 0.001.

As shown in Fig. 8, immunoblotting showed that LPS treatment significantly upregulated the phosphorylation of TAK1 and JNK compared with that in the saline group (*P* < 0.05). Notably, the phosphorylation levels of TAK1 and JNK were markedly enhanced in the context of TIPE2 knockout; in contrast, overexpression of TIPE2 in DCs significantly weakened the phosphorylation of TAK1 and JNK in comparison to that in the WT-LPS group (*P* < 0.05).

## Discussion

Sepsis is now a severe clinical syndrome and a key concern in the field of public health, which is associated with high mortality and, for the survivors, long-term morbidity [5, 9–11, 32]. The current paradigm with respect to the host immune response to infections in sepsis is still controversial [33, 34]. Evidently, sepsis-induced immunosuppression has been proven to be related to deleterious outcomes in septic patients. Numerous studies have reported the critical role of DCs in orchestrating immune responses throughout the course of sepsis, and these cells play an essential interface between innate and adaptive immunity [12, 13, 35–37]. Functional impairment of DCs has been widely reported, and is responsible for the unbalanced and disrupted host response to infection [38–40].

TIPE2 plays a critical role in regulating the inflammatory response and maintaining immune homeostasis [22, 41]. Wang et al. [42] found that TIPE2 markedly attenuated corneal inflammation induced by *Pseudomonas aeruginosa* infection by inhibiting the infiltration of inflammatory cells. Genetic deficiency in TIPE2 resulted in significant immunopathological changes, including splenomegaly, leukocytosis, hypersensitivity to Toll-like receptor (TLR) and T-cell receptor (TCR) actuation, and cytokine overproduction, due to the proapoptotic effect of TIPE2 via the negative regulation of the activating protein (AP)-1 and nuclear factor (NF)-κB pathways, as well as the interaction with Caspase-8 and FasL [22].

It is evident that autophagy serves as a major modulator in several biological processes of DCs relevant to the immune function of DCs [21, 43, 44]. In this study, we found that the autophagy activity in DCs was most evident after LPS treatment for 12 h. Moreover, we observed that autophagic activity in TIPE2^−/−^ DCs was significantly enhanced compared to that in WT DCs challenged by LPS in vitro. Conversely, overexpression of TIPE2 restrained the autophagy levels remarkably in TIPE2^+/+^ DCs, implying that TIPE2 could negatively regulate autophagy in DCs in vitro. Furthermore, in the CLP model, the autophagic activity of DCs was markedly elevated in TIPE2^−/−^ mice; in contrast, DC autophagic activity was significantly suppressed after the septic challenge. These results further confirm the inhibitory effect of TIPE2 on DC autophagy in the setting of sepsis.

Next, we further sought to examine whether TIPE2-mediated regulation of DC immune function was dependent on autophagic activity. Consistent with a previous study [24], it was noted that TIPE2 exerted a negative regulation on the LPS-mediated immune response of DCs in this study, as evidenced by the dramatical functional deterioration in TIPE2^+/+^ DCs and functional improvements in TIPE2^−/−^ DCs after LPS stimulation in vitro. Interestingly, the silencing of Beclin-1 gene obviously weakened the functions of TIPE2^−/−^ DCs by reversing the effect of the absence of TIPE2 on autophagy. Similarly, it was observed that TIPE2^+/+^ DC functions were notably improved due to Beclin-1 overexpression-mediated augmentation of autophagy. In addition, we further verified these results in CLP mice. Therefore, our data suggest that TIPE2 can impair the immune function of DCs by downregulating the autophagic activity of DCs following septic insult.

Many studies have indicated that the mitogen-activated protein kinase (MAPK)/JNK pathway is involved in the regulation of various forms of autophagy, such as starvation-induced autophagy [45], neurotoxicity-induced autophagy [46], and cytotoxicity-induced autophagy [47]. TAK1 is an upstream kinase of the JNK pathway that activates JNK and p38 MAPK by activating MKK3, MKK4, MKK6, and MKK7 MAPKKs [48]. Other studies suggested that TIPE2 could coprecipitate with TAK1 in the cytoplasm of RAW264.7 cells or human corneal epithelial cells and functions as a negative regulator of TAK1 phosphorylation [42, 49]. Given the multipotent effects of TIPE2, it is reasonable for us to hypothesize that TIPE2 inhibits autophagy in DCs by downregulating the TAK1-JNK pathway, although it remains largely unknown whether the TAK1-JNK signaling pathway involves in the regulation of DC autophagy.

Herein, we addressed whether TIPE2 suppressed the LPS-stimulated activation of TAK1-JNK axis in this study. It was noticed that LPS-induced phosphorylation of TAK1 and JNK in TIPE2^+/+^ DCs was strongly inhibited, while the phosphorylation levels of TAK1 and JNK were markedly enhanced in TIPE2^−/−^ DCs, which supported the conclusion that TIPE2 displayed an inhibitory impact on TAK1-JNK signaling pathway. Moreover, our data revealed that the autophagy was distinctly depressed due to the blockade of the TAK1-JNK pathway. Of note, the autophagic response remained weak although obvious activation of TAK1 induced by LPS was observed after we perturbed the JNK phosphorylation, indicating that the TAK1-JNK pathway might serve as a crucial regulator of autophagy in DCs evoked by LPS. Thus, TIPE2 appears to be a potent repressor of autophagy in DCs via downregulation of the TAK1-JNK pathway.

In summary, to the best of our knowledge, this work is the first investigation to explore the relationship between TIPE2 and autophagy in DCs in sepsis. We demonstrate that TIPE2 exerts a negative effect on autophagy and further impairs the immune function of DCs. The TAK1-JNK pathway is required in TIPE2-mediated regulation of autophagy in DCs, thereby uncovering a novel immunoregulatory mechanism of TIPE2. Although the therapeutic potential of TIPE2 in the modulation of the inflammatory response has attracted considerable interest [41, 42, 50], there is still much work to be done before clinical application. Taken together, our results suggest that TIPE2 can exacerbate sepsis-induced DCs immune dysfunction by restricting autophagy via the TAK1-JNK pathway. However, the more precise mechanism by which TIPE2 affects immune functions of DCs in sepsis is still needed to be elucidated, which is the main subject of our forthcoming study.

## Supplementary information

Supplementary legends

Materials and methods

Supplementary Figure S1

## Data Availability

The datasets used and analyzed during the current study are available from the corresponding author on reasonable request.
