# Supplementary material for: TNF-α-induced protein 8-like 2 negatively regulates the immune function of dendritic cells by suppressing autophagy via the TAK1/JNK pathway in septic mice
Source: Cell Death Dis. 2021 Oct 30;12(11):1032. doi: 10.1038/s41419-021-04327-x (PMC8557212; doi:10.1038/s41419-021-04327-x)
Supplement: Supplementary file 1 — Supplementary legends [file 41419_2021_4327_MOESM1_ESM.docx]

**Supplementary legend**

**Supplementary Figure S1.** **Stimulation with LPS enhanced autophagy activity of DCs *in vitro*.** Mice splenic DCs were treatment by LPS *in vitro* at a dose of 1 μg/ml for various intervals (0, 3, 6, 12 and 24h, respectively). Total cell protein was extracted, and levels of autophagy related proteins were determined by Western blotting, respectively. β-actin served as the internal standard. Values of three independent experiments were represented as mean ± SD (n=5 in each group). Statistical significances: compared with the 12h group, ^**^*P*<0.01; ^***^*P*<0.001.
