# Supplementary material for: TNF-α-induced protein 8-like 2 negatively regulates the immune function of dendritic cells by suppressing autophagy via the TAK1/JNK pathway in septic mice
Source: Cell Death Dis. 2021 Oct 30;12(11):1032. doi: 10.1038/s41419-021-04327-x (PMC8557212; doi:10.1038/s41419-021-04327-x)
Supplement: Supplementary file 2 — Materials and methods [file 41419_2021_4327_MOESM2_ESM.docx]

**Materials and methods**

**Animals**

Experiments were performed on six-week-old male C57BL/6J mice (weight range 20-25 g), which were provided by the Institute of Laboratory Animal Science, Peking Union Medical College, Beijing, China. TIPE2 gene knockout (TIPE2^-/-^) and knock-in (TIPE2^+/+^) mice on a C57BL/6J background were constructed by the Nanjing Biomedical Research Institute of Nanjing University, Nanjing, China. Animals were randomly divided into different experimental groups or control groups. All mice (4-5 mice per cage) were maintained in SPF conditions with a 12-hour (h) dark-light cycle at a temperature of 22-25 ℃. The animals were provided with laboratory chow and water *ad libitum*.

**Generation of TIPE2^-/-^ and TIPE2^+/+^ mice**

The mice were housed at the Nanjing Biomedical Research Institute of Nanjing University, Nanjing, China (Project Nos. XM707413, XM707414). All procedures were preapproved by the Institutional Animal Care and Use Committee at Nanjing University. The TIPE2^-/-^ model was created by CRISPR/Cas9 (D10A)-mediated genome engineering. A pair of single-guide RNAs (sgRNAs) targeting exon 2 of the TIPE2 gene were constructed and transcribed *in vitro*. Cas9 (D10A) messenger RNA and sgRNAs were co-injected into 0.5-day-old C57BL/6J mouse zygotes.

To establish the TIPE2^+/+^ model, the transcript *Tnfaip812-201* (ENSMUST00000013851.3) was selected for use in the recommended strategy. The *H11-CAG-Tnfaip812-P2A-EGFP-polyA* knock-in mouse model was generated *via* the CRISPR/Cas9 system. Cas9 mRNA, sgRNA and donor RNA were co-injected into zygotes. The sgRNA directs Cas9 endonuclease cleavage at the *H11* locus and creates a double-strand break. Such breaks were repaired and resulted in the insertion of *CAG-Tnfaip812-P2A-EGFP-polyA* in the *H11* locus.

**Reagents**

CD4 (L3T4) microbeads, CD11c (N418) microbeads, and phycoerythrin (PE)-Cyanine 5-conjugated anti-mouse major histocompatibility complex (MHC)-II were purchased from eBioscience (San Diego, CA). PE-conjugated anti-mouse CD86, allophycocyanin (APC)-conjugated anti-mouse CD80 and fluorescein isothiocyanate (FITC)-conjugated goat anti-mouse CD11c were purchased from Miltenyi Biotec (GmbH, Bergisch Gladbach, Germany). Lipopolysaccharide (LPS, *E. coli* O127:B8) and Triton X-100 were obtained from Sigma-Aldrich, (St Louis, MO). Roswell Park Memorial Institute (RPMI) 1640, fetal calf serum (FCS), penicillin, and streptomycin were purchased from TianRunShanda Biotech Co. Ltd. (Beijing, China). Phosphate-buffered saline (PBS) was purchased from TBD Science (Tianjin, China). TIPE2 polyclonal antibodies and R-PE-conjugated goat anti-rabbit IgG (H+L) antibodies were purchased from Proteintech Inc. (Rosemont, MN). FITC-mouse anti-rabbit IgG was purchased from Santa Cruz Biotechnology (Santa Cruz, CA). SQSTM1/p62 antibodies, Beclin-1 (D40C5) rabbit monoclonal antibodies, LC3B (D11) XP-rabbit monoclonal antibodies, c-Jun N-terminal kinase (JNK) antibody, phospho-JNK (Thr183/Tyr185) rabbit monoclonal antibodies, transforming growth factor-β (TGF-β)-activated kinase-1 (TAK1) (D94D7) rabbit monoclonal antibodies and phospho-TAK1 (Ser412) antibodies were purchased from Cell Signaling Technology (Danvers, MA). Bafilomycin A1, 3-Methyladenine (3-MA), [rapamycin](javascript:;) (Rap), SP600125 (HY-12041), and Takinib (HY-103490) were purchased from MedChemExpress (Shanghai, China).

**Cecal ligation and puncture model**

The cecal ligation and puncture (CLP) mouse model is a commonly used animal model used to reproduce polymicrobial sepsis. After anesthesia (5% chloral hydrate), the mice were fixed on the operation table, and then the abdominal area was disinfected. An incision (1.0-1.5 cm) was made along the midline of the abdomen, and then the peritoneum was opened to identify and expose the cecum. The cecum was ligated in the middle and punctured twice using a 21-gauge needle to induce sepsis. Next, the cecum was placed back in the abdominal cavity. Finally, 1 ml of 0.9% normal saline was injected subcutaneously. Sham-operated mice underwent the same surgical procedure without the ligation and perforation steps.

**Isolation of splenic DCs and T lymphocytes**

Under aseptic conditions, murine spleens were removed from the abdominal cavity and washed twice using precooled PBS. Mononuclear cells were isolated from the spleens, lymphocytes were isolated using a CD4^+^ T cell isolation kit (Miltenyi Biotec GmbH, Bergisch Gladbach, Germany), and DCs were isolated using a CD11c^+^ dendritic cell isolation kit (Miltenyi Biotec GmbH, Bergisch Gladbach, Germany) with a positive selection MS column according to the manufacturer’s instructions. The isolated DCs and CD4^+^ T lymphocytes were pelleted by centrifugation (at 250 g for 15 min), the supernatant was discarded, and the selected cells were cultured in RPMI 1640 with 10% heated-inactivated FCS 100 U/ml penicillin and 100 μl/ml streptomycin at 5% CO_2_ and 37°C in a humidified incubator or were used for experiments.

**RNA interference**

Small interference RNA to Beclin-1 (siRNA) or over-expression RNA to Beclin-1 (LV RNA) was generated by Genchem Co., Shanghai, China. To knockdown or up-regulate Beclin-1 expression, DCs were introduced with recombinant lentiviruses that carry the Beclin-1-siRNA or Beclin-1-LV-RNA. Transfection of recombinant lentiviruses was performed according to the manufacture’s instruction. The transduction efficiency for DCs *in vitro* was greater than 60%. After transfection for 3 days, the efficiency of knockdown or overexpression was determined by Western blotting for Beclin-1 expression.

**Flow cytometric analysis of costimulatory molecules on DCs**

DCs were isolated from C57BL/6J mouse spleens [wild-type (WT), TIPE2^-/-^, and TIPE2^+/+^]. *In vitro*, DCs were stimulated with LPS for 12 h, and normal DCs were used as the controls. *In vivo*, DCs were harvested from septic mice, and negative controls were designed. Cells (5×10^5^) in 100 μl of PBS supplemented with 5% FCS and 0.1% sodium azide (staining buffer) were incubated for 30 min at 4°C with PerCP/Cyanine 5.5 anti-mouse CD80, APC anti-mouse CD86, FITC anti-mouse I-A/I-E, and FITC-conjugated anti-mouse CD11c antibodies (BioLegend, San Diego, CA). Then, the cells were fixed with 1% paraformaldehyde or analyzed by flow cytometry within 1 h using a FACScan (BD Biosciences, Mountain View, CA).

**Laser scanning confocal microscopy**

TIPE2 expression and autophagic activity in splenic DCs were observed by laser scanning confocal microscopy (LSCM). Cells (2×10^6^) were harvested and subjected to LPS stimulation for 12 h. After being washed twice with PBS, the cells were permeabilized with 0.5% Triton X-100 or digitonin and fixed with 4% formaldehyde for 1 h after being washed three times with PBS. The cells were blocked with 5% bovine serum albumin for 1 h at room temperature in the dark and incubated with primary antibodies at 4 °C overnight and with FITC- and PE-conjugated goat antibodies for 1 h. The cell nuclei were stained with 4',6-diamidino-2-phenylindole dihydrochloride (DAPI). Finally, images were acquired under a fluorescence microscope. The samples were postfixed with 1% osmium tetroxide, dehydrated sequentially with ethanol and embedded in epoxy resin.

**Transmission electron microscopy**

DCs were fixed in 4% glutaraldehyde containing 0.1 M cacodylate buffer for 1 h at 4°C and then fixed in 1% osmium tetroxide for 1 h at room temperature. The samples were embedded in epoxy resin after being dehydrated through a graded ethanol series. Ultrathin sections were prepared with a diamond knife and stained with uranyl acetate-lead citrate. DC autophagosomes were identified and counted by using a Hitachi H-7500 transmission electron microscope (Hitachi, H-7500, Japan).

**Western Blotting Analysis**

Cells (8×10^6^) were collected and washed twice using ice-cold PBS, and protein was extracted using RIPA buffer containing a proteinase inhibitor cocktail and quantified with a Bio-Rad protein assay (Bio-Rad Laboratories, Hercules, CA). The cell lysate was loaded into the EP tube (30 μg/30 μl) and boiled at 95°C for 5 min after being mixed with SDS-loading buffer. Equal amounts of protein were separated by sodium dodecyl sulfate-polyacrylamide gel electrophoresis and then transferred electrophoretically onto polyvinylidene difluoride membranes. Blotting was performed according to standard procedures with primary antibodies against TIPE2, LC3 I / II, p62, Beclin-1, TAK1, p-TAK1, JNK, p-JNK and β-actin overnight, followed by incubation with secondary antibodies conjugated with horseradish peroxidase. Immunoreactivity was visualized by an ECL detection system (Amersham Biosciences, Uppsala, Sweden). Protein levels were quantified by densitometric analysis.

**Enzyme-linked immunosorbent assay**

To assess the release of cytokines associated with DC maturation, IL-12 and TNF-α levels in supernatants of splenic DCs or peripheral blood were measured by enzyme-linked immunosorbent assay (ELISA) using mouse TNF-α/IL-12 p40 simple step ELISA kits (Excell Inc., Shanghai, China). To assess the types of T helper responses, the levels of [interferon](javascript:;) (INF)-γ and IL-4 in the culture medium of CD4^+^ T cells cocultured with DCs were analyzed by ELISA kits according to the manufacturer’s instructions (Excell Inc., Shanghai, China). The plates were read in a microplate reader (Spectra MR, Dynex, Richfield, MN).

**Carboxyl fluorescein succinyl ester staining**

CD3 was prepared at a concentration of 5 μg/mL, and 5 μL of the CD3 solution was added to a 96-well plate and incubated at 37°C for 120 min. The isolated CD4^+^ T cells were stained with carboxyl fluorescein succinyl ester (CFSE, 5 μM) and incubated at room temperature in the dark for 20 min. A 5-fold volume of PBS was added to terminate the staining, and the cells were centrifuged. CD4^+^ T cells were resuspended in medium supplemented with CD28 and incubated for 24 h. DCs were treated with LPS or saline and cocultured with CD4^+^ T cells at a ratio of 1:100 for 72 h.
