# Supplementary figures and images for: TNF-α-induced protein 8-like 2 negatively regulates the immune function of dendritic cells by suppressing autophagy via the TAK1/JNK pathway in septic mice
Source: Cell Death Dis. 2021 Oct 30;12(11):1032. doi: 10.1038/s41419-021-04327-x (PMC8557212; doi:10.1038/s41419-021-04327-x)

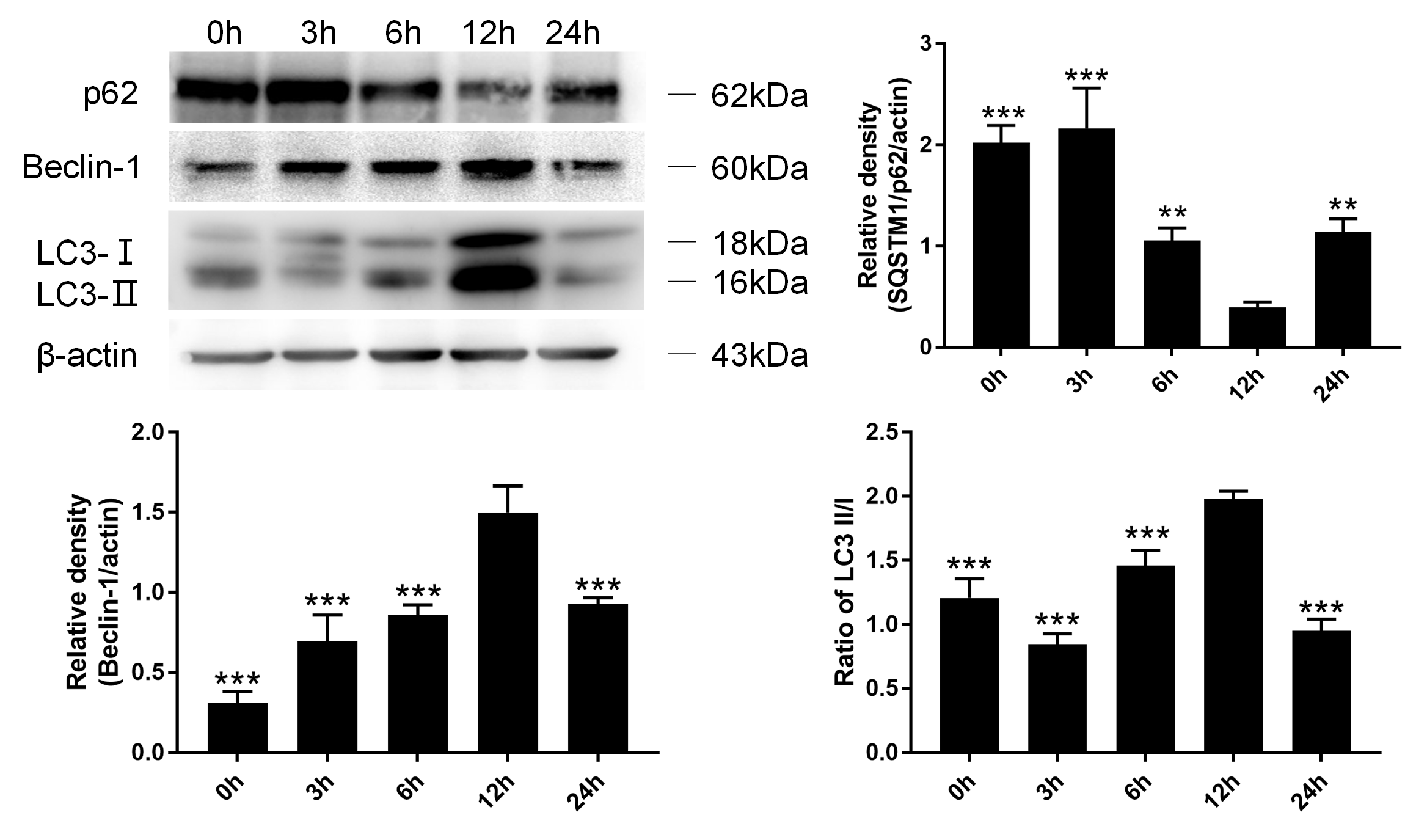

Supplement: Supplementary file 3 — Supplementary Figure S1 [file 41419_2021_4327_MOESM3_ESM.tif]
